# Outcomes of Root Canal Treatment in Patients With Autoimmune Disease: A Retrospective Case–Control Study

**DOI:** 10.1111/iej.70174

**Published:** 2026-05-04

**Authors:** Seoyeon Lee, Euiseong Kim, Hoiin Jung, Hyejin Kim, Sunil Kim

**Affiliations:** ^1^ Department of Conservative Dentistry Veterans Health Service Medical Center Seoul South Korea; ^2^ Microscope Center, Department of Conservative Dentistry and Oral Science Research Center Yonsei University College of Dentistry Seoul South Korea; ^3^ Department of Preventive Dentistry & Public Oral Health Yonsei University College of Dentistry Seoul South Korea

**Keywords:** apical periodontitis, autoimmune disease, clinical outcome, inflammatory bowel disease, psoriasis, rheumatoid arthritis, root canal treatment, systemic disease

## Abstract

**Aim:**

The purpose of this study was to compare the outcome of nonsurgical root canal treatment (RCT) in patients with autoimmune diseases (AD) with the outcome in patients without AD. The null hypothesis was: there is no difference in the outcomes of RCT regardless of the patient group. Results were also compared among AD subgroups: inflammatory bowel disease (IBD), rheumatoid arthritis (RA), and psoriasis (Ps).

**Methodology:**

Data were obtained from a database using codes corresponding to the National Health Insurance Dental Fee Schedule. Patients diagnosed with AD who received primary RCT at the Veterans Health Service Medical Center from 2010 to 2022 formed the study group. Controls were matched using propensity score matching for age, sex, arch type, and tooth type. Preoperative periapical lesions, canal filling quality, and RCT outcomes were assessed through radiographs, including panoramic, periapical, and cone‐beam computed tomography images. Outcomes were evaluated at approximately 1 year and at maximum follow‐up. Chi‐square tests and logistic regression were used to evaluate associations between AD and RCT success.

**Results:**

The study included 203 ad patients (317 teeth) and 203 controls (312 teeth). At 1 year, success rates were 69.4% in AD patients and 73.7% in controls (*p* = 0.268). Within the AD group, success rates were 64.6% for IBD, 75.3% for RA, and 73.2% for Ps. Patients with IBD showed only statistical significance (*p* = 0.025). The mean observation duration for the maximum follow‐up was approximately 50 months. Long‐term success rates were 61.5% for AD and 66.0% for controls (*p* = 0.207). Multivariate logistic regression analysis indicated that arch type, tooth type, and the presence of preoperative apical lesions were significant factors influencing the outcome of RCT. Comparing the 1‐year and long‐term outcomes, IBD, RA, and Ps showed a similar trend of decreased success rates, although the differences were not statistically significant.

**Conclusions:**

RCT success rates in patients with AD were comparable to those of controls. Multivariable analysis revealed that clinical factors were the primary predictors of success, rather than the presence of AD. Therefore, high‐quality clinical procedures are the critical factor for favorable outcomes in AD patients.

## 1 Introduction

The relationship between systemic health and the outcomes of root canal treatment (RCT) has emerged as a significant focus in endodontics. According to Aminoshariae et al., several systemic diseases were found to be correlated with the outcome of endodontic treatment (Aminoshariae et al. 2017). Diabetes mellitus has been reported to have an adverse influence on the healing of endodontically treated teeth with preoperative infections, suggesting its potential role as a modifying factor in treatment outcomes. (Fouad and Burleson 2003; Lima et al. 2013). In addition, previous studies have reported reduced survival of endodontically treated teeth in patients with diabetes or hypertension (Mindiola et al. 2006). Given the similarities in pathogenic mechanisms, a potential correlation between apical periodontitis (AP) and systemic diseases may also be plausible.

Autoimmune diseases (AD) encompass a diverse group of disorders with varying clinical manifestations, yet they share a common aetiology characterized by a self‐reactive immune response (Wang Wang et al. 2015). According to The Global Burden of Diseases, Injuries, and Risk Factors Study (GBD), the global prevalence of AD has been increasing (Temporal trends in the prevalence of autoimmune diseases from 1990 to 2019). Inflammatory Bowel Diseases (IBD) encompass chronic, immune‐mediated inflammatory conditions of the gastrointestinal tract, primarily categorized into Ulcerative colitis and Crohn's disease (Baumgart and Carding 2007). IBD emerges when the equilibrium between proinflammatory and anti‐inflammatory pathways is disturbed (Pastorelli et al. 2010; Piras et al. 2017). Rheumatoid Arthritis (RA) is a persistent autoimmune condition affecting the joints, marked by pain, inflammation, and progressive tissue destruction (Smolen et al. 2020). The exact pathogenesis of RA remains unclear, yet it is believed to be mediated by multiple interacting factors, including autoimmunity, infectious agents, and genetic factors (McInnes and Schett 2011). Furthermore, psoriasis (Ps) is characterized as a chronic inflammatory disorder predominantly affecting the cutaneous tissue (Raychaudhuri and Gross 2000), strongly linked to genetic susceptibility and autoimmune traits (Harden et al. 2015). In recent years, growing interest has emerged regarding the relationship between AP and systemic disease, leading to the development of the concept of endodontic medicine (Segura‐Egea et al. 2015).

Despite the increasing trend of autoimmune diseases, there is a lack of clear evidence reporting a correlation between AD and dental diseases. Previous investigations into systemic diseases in the context of endodontic treatment outcomes have predominantly concentrated on tooth survival rather than the process of apical healing (Laukkanen et al. 2019). There were few previous studies comparing patients with and without autoimmune diseases in the light of RCT outcomes. The present study focused on the association between AD and AP at the level of clinical outcomes. This study aims to compare the outcomes of nonsurgical RCT in patients with AD—IBD, RA, and Ps—with the outcome in patients not affected by AD. The null hypothesis is: there is no difference in the outcomes of RCT regardless of the patient group.

## 2 Material & Methods

### 2.1 Ethical Considerations

An evaluation was conducted of the medical and dental records of patients who attended the medical and dental clinics at the Veterans Health Service Medical Center (Seoul, South Korea) for a dental evaluation from January 2010 to December 2022. The personal data were electronically anonymized prior to analysis. Given that only fully anonymized retrospective data were utilized, this study was a non‐interventional observational study conducted under a standardized non‐experimental protocol. Accordingly, Institutional Review Board (IRB) approval was officially obtained (IRB file No. 2025–03‐001).

### 2.2 Data Collection & Case Selection

#### 2.2.1 The Study Group (With Autoimmune Disease)

Patients who were diagnosed with AD and received RCT at the Veterans Health Service Medical Center from January 2010 to December 2022 formed the target population. The study consisted of both males and females who were diagnosed with AD, such as IBD, RA, or Ps. A total of 203 participants with 317 teeth were included in the AD study group. This patient group was further divided into three subgroups based on the specific disease: IBD (*n* = 147), RA (*n* = 73), and Ps (*n* = 97). Table 1 shows descriptive names of diagnosis of the autoimmune disease.

**TABLE 1:** Autoimmune disease diagnoses and number of patients (*n*).

Disease	Diagnosis	Number of patients	Reimbursement code according to the National Health Insurance system
Inflammatory Bowel Disease (IBD)	Inflammatory bowel disease Ulcerative colitis with pancoilitis Ulcerative colitis with proctitis Ulcerative colitis Ulcerative coilitis, unspecified Crohn's disease of small intestine (mild, moderate, severe) Crohn's disease of small intestine, unspecified Crohn's disease of large intestine (mild, moderate, severe) Crohn's disease of large intestine, unspecified Crohn's disease. Unspecified	109 1 10 10 17 0 0 0 0 0	K529 K510 K512 K518 K519 K5000—K5002 K5009 K5010—K5012 K5019 K5099
Rheumatoid Arthritis (RA)	Other seropositive rheumatoid arthritis Rheumatoid arthritis with involvement of other organs and systems Mild other specified rheumatoid arthritis, multiple sites Moderate other specified rheumatoid arthritis, multiple sites Severe other specified rheumatoid arthritis, multiple sites Unspecified other specified rheumatoid arthritis, multiple sites	7 66 0 0 0 0	M0529 M0531—M0539 M06800 M06801 M06802 M06809
Psoriasis (Ps)	Psoriasis Mild psoriasis vulgaris Moderate psoriasis vulgaris Severe psoriasis vulgaris Psoriasis vulgaris	97 0 0 0 0	L409 L4000 L4001 L4002 L4003

#### 2.2.2 The Control Group (Without Autoimmune Disease)

A total of 14 572 patients with 15 489 teeth were included in the total control group (Table 2). Due to the impracticality of reviewing the large volume of data from patients treated during the same period, a propensity score matching (PSM) approach was employed to establish a representative control group. The type of tooth was recorded as molars, premolars, or incisors. The type of arch was recorded as maxilla or mandible. Considering that preferred materials and first‐line treatment options may evolve over time, treatment dates were matched across groups to minimize temporal bias. The final control group was established through a second propensity score matching that incorporated exclusion criteria and follow‐up period as matching variables.

**TABLE 2:** The demographic information of the study.

	Total control, *N* (%)	Control, *N* (%)	AD, *N* (%)
Patient	14 572	203 (50)	203 (50)
Teeth	15 489	312 (49.2)	317 (50.8)
IBD			147 (46.4)
RA			73 (23.0)
Ps			97 (30.6)
Sex			
Male	11 122 (76.6)	285 (91.4)	288 (90.9)
Female	3450 (23.4)	27 (8.6)	29 (9.1)
Age	77.68 ± 8.01	71.88 ± 8.02	72.18 ± 6.20
Radiographs at 1‐year			
Panorama		243 (77.9)	163 (51.4)
PA		34 (10.9)	71 (22.4)
CBCT		35 (11.2)	83 (26.2)
Radiographs at long‐term			
Panorama		227 (72.8)	181 (57.1)
PA		55 (17.6)	54 (17.0)
CBCT		30 (9.6)	82 (25.9)
Tooth type			
Maxillary incisors	2537 (16.4)	53 (17.0)	48 (15.1)
Maxillary premolars	2447 (15.8)	42 (13.5)	43 (13.6)
Maxillary molars	3094 (20.0)	62 (19.9)	65 (20.5)
Mandibular incisors	1829 (11.8)	30 (9.6)	46 (14.5)
Mandibular premolars	2262 (14.6)	59 (18.9)	47 (14.8)
Mandibular molars	3320 (21.4)	66 (21.1)	68 (21.6)
Presence of preoperative periapical lesion			
		105 (33.7)	129 (40.7)
IBD			59 (40.1)
RA			26 (35.6)
Ps			44 (45.4)
Quality of canal filling			
Adequate		302 (96.8)	299 (94.3)
Underfilling		9 (2.9)	14 (4.4)
Overfilling		1 (0.3)	4 (1.3)

Abbreviations: AD, autoimmune disease; CBCT, cone beam computed tomography; IBD, inflammatory bowel disease; Ps, psoriasis; PA, periapical radiographs; RA, rheumatoid arthritis.

The inclusion and exclusion criteria were as follows:

Inclusion Criteria:

Patients underwent primary RCT and had representative variable matching to those of the study group: age, gender, arch type, tooth type, timing of the treatment.

Teeth with sufficient follow‐up data (minimum 1‐year post‐treatment).

Exclusion Criteria:

Teeth extracted for non‐endodontic reasons within the follow‐up period, most commonly involving severe periodontal involvement or restorative failure.

### 2.3 Data Collection and Acquisition of Samples

All data were anonymized before extraction, and patient privacy was strictly protected. A programmed interface enabled retrieval of tooth‐specific treatment histories through database queries. The database records both reimbursable treatment codes defined by the National Health Insurance Dental Fee Schedule (HIRA) and non‐reimbursable fee codes regulated by the Ministry of Health and Welfare. For this study, all data related to the patients who were diagnosed with AD, the diagnosis codes of which are listed in Table 1, at the medical clinic from January 2010 until December 2022 were included in the analysis. Data regarding the diagnosed disease, the date of diagnosis, and the clinician who made the diagnosis were obtained. In this database, cases that received RCT were identified by the code CDA0000090 which is the code for root canal filling. As the corresponding code applies to both primary and non‐primary root canal treatments (RCTs), individual records were reviewed to identify and exclude cases of retreatment.

Other variables that could only be obtained from radiographic images were manually reviewed and recorded. All radiographic images taken at this institution were stored in the PACS system (INFINITT Healthcare Co. Ltd., Seoul, Korea), and the radiographic records were retrieved using a list that contained the corresponding invoice codes and dates of service. Within the framework of the National Health Insurance reimbursement system, periapical radiographs are almost invariably taken on the date of root canal filling. To obtain valid information from the records available after the completion of root canal filling, the following parameters were investigated and documented: the date of the dental radiograph record closest to the 1‐year mark post‐treatment, the type of dental radiographs taken at the 1‐year mark, the most recent date of the dental radiograph record, and the type of dental radiographs taken at the most recent visit. Periapical radiolucency and the quality of root canal filling were evaluated based on the radiographic images obtained.

**FIGURE 1:**
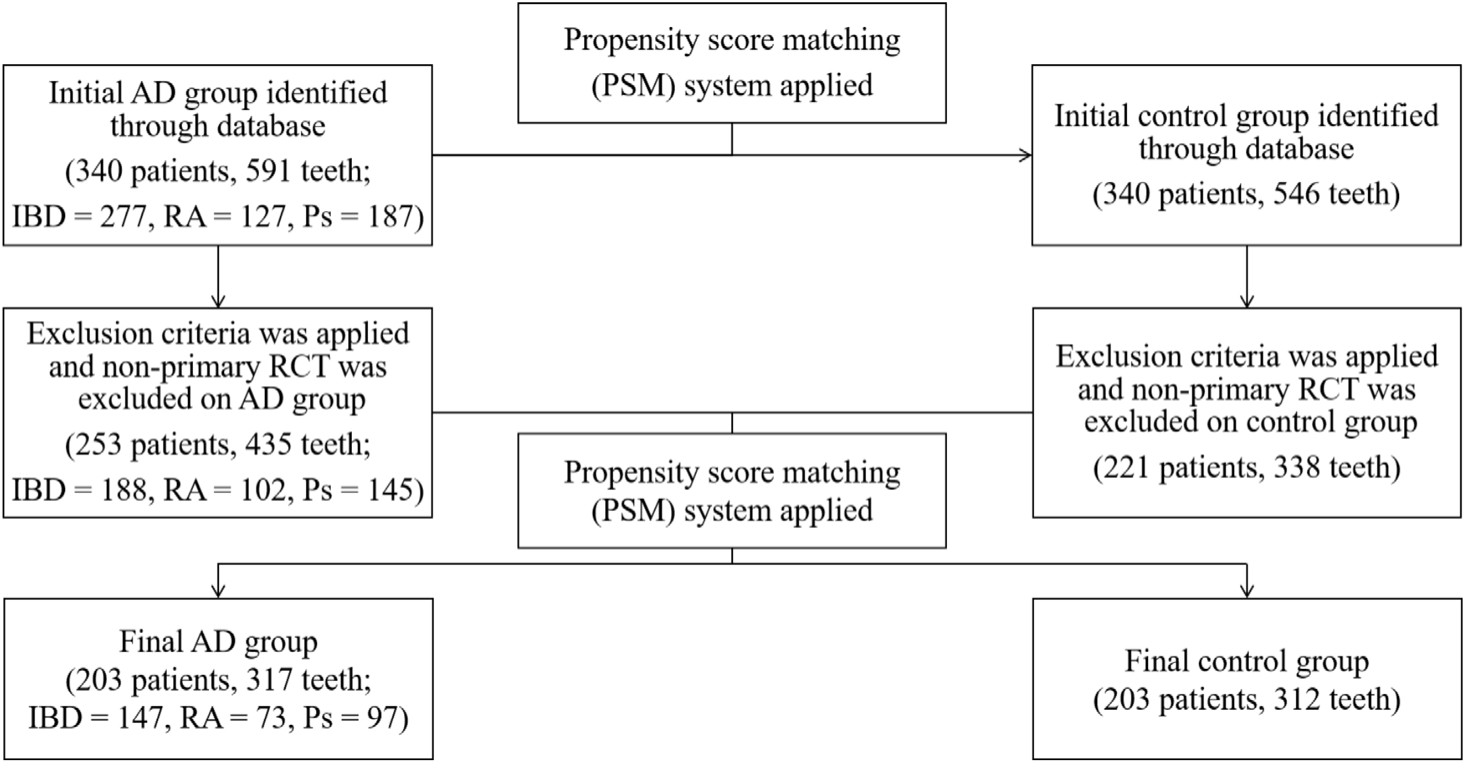
Flowchart of patient and tooth selection for the study. AD, autoimmune disease; IBD, inflammatory bowel disease; Ps, psoriasis; RA, rheumatoid arthritis; RCT, root canal treatment.

Using the employed database, an initial AD group was established through a query of patients who had been diagnosed with ADs, the diagnostic codes of which are listed in Table 1, at the medical clinic between January 2010 and December 2022. Cases that underwent RCT were identified using the reimbursement code CDA0000090, which corresponds to root canal filling. Through this identification process, an initial AD group comprising 340 patients and 591 teeth (IBD = 277, RA = 127, Ps = 187) was obtained. As the reimbursement code encompasses both primary and non‐primary RCTs, the initial AD group included cases of both types. A control group, which likewise included both primary and non‐primary RCT cases, was established through the same database identification process. A total of 14 572 patients with 15 489 teeth were included in the total control group. Using the propensity score matching (PSM) system, an initial control group with comparable age, sex, arch type, tooth type, and timing of the treatment was established, resulting in 340 patients and 546 teeth. After applying the exclusion criteria and removing cases that were not primary RCTs, 253 patients and 435 teeth (IBD = 188, RA = 102, Ps = 145) remained in the AD group. The same exclusion process was applied to the control group, yielding 221 patients and 338 teeth. After a second round of propensity score matching, the final AD group consisted of 203 patients and 317 teeth (IBD = 147, RA = 73, Ps = 97). The overall process of patient and tooth selection is summarized in Figure 1.

### 2.4 Treatment Protocol

The primary endodontic treatments were performed by endodontists and dental residents specializing in endodontics. While the treatments generally adhered to the guidelines outlined in the American Association of Endodontists' (AAE) Guide to Clinical Endodontics, they were not strictly bound to a single protocol. All procedures were carried out under rubber dam isolation to prevent saliva contamination. The root canal apex was located using an electronic apex locator (Root ZX, Root ZX II by Morita, Japan), with the working length confirmed through both electronic apex locator (EAL) readings and periapical radiographs. The apical third was prepared using Profile NiTi files (Dentsply Tulsa Dental, OK, USA), the rotary Race Evo instrument system (FKG Dentaire SA, La Chaux‐de‐Fonds, Switzerland), and ProTaper NiTi files (Dentsply Tulsa Dental, Tulsa). Sodium hypochlorite (2.5%–5%) was the primary irrigant, and in some cases, EDTA (17% ethylenediamine‐tetra‐acetic acid) or 2% chlorhexidine gluconate were used optionally. Calcium hydroxide paste (Calcipex II, Nishika, Tokyo, Japan) was applied as an intracanal medicament between appointments. Root canals were filled with gutta‐percha, along with either an epoxy resin‐based sealer (AH‐26, AH Plus, Dentsply Sirona, York, PA, USA), a calcium hydroxide‐based sealer (Sealapex, Kerr, Orange, CA, USA), or a zinc oxide‐eugenol‐based sealer (Tubli‐seal, Kerr, Orange, CA, USA), using a technique selected by the operator.

### 2.5 Radiographic Evaluation

All radiographs were digital. Dental radiographs were assessed using Trophy DICOM software (version 6.4; Carestream Dental, Croissy‐Beaubourg, France) on a 15‐in. HP monitor, with brightness and contrast adjusted as required to ensure optimal image evaluation.

The quality of canal filling and periapical conditions were independently assessed by two examiners using the Periapical Index (PAI) scoring system, as described by Ørstavik et al. (Orstavik et al. 1986) and the Cone Beam Computed Tomography Periapical Index (CBCT‐PAI) system, proposed by Estrela et al. (Estrela et al. 2008). The evaluation was carried out by a postgraduate student in endodontics (S. L.) and an endodontic specialist (S. K.). To minimize bias, the examiners were blinded to group allocation and were unaware of whether each radiograph correspond to the study or control group. Two blinded examiners separately performed visual analysis of all the images. All CBCT cross sections and periapical radiographs were randomized before evaluation. The level of intraobserver agreement was assessed by weighted kappa statistics in 20% of the sample repeated 1 month after initial evaluation, and the level of interobserver agreement was calculated over the entire data set. Interexaminer agreement for radiographic evaluations at the final follow‐up was assessed by using Cohen kappa statistic (κ = 0.90). If there were discrepancies in the radiographic evaluation, the two examiners reached a consensus through discussions.

The quality of canal fillings was evaluated using radiographic images. An obturation was considered adequate if it terminated within 2 mm of the radiographic apex on periapical radiographs. Cases with greater deviations—defined as fillings that extended beyond the apex or ended more than 2 mm short of the apex—were considered inadequate. Based on the direction of deviation, these cases were classified as underfilled or overfilled. The quality of root canal filling was further assessed in terms of lateral seal. A filling was considered adequate when no voids were present along the entire canal length and a homogeneous radiopaque appearance was observed. The presence of voids or radiographic irregularities in the coronal half and/or apical half of the canal was recorded as underfilled. Cases in which a canal was missing were also regarded as underfilled.

The Periapical Index (PAI) is a 5‐point ordinal scale used to assess periapical status, with a score of 1 representing a healthy state and a score of 5 indicating severe AP with signs of exacerbation. The guidelines for scoring cases using the PAI are as follows: (1) Identify the reference radiograph that most closely matches the periapical area under study and assign its score to the relevant root. (2) In cases of uncertainty, the higher score should be applied. (3) For teeth with multiple roots, the final score is determined based on the root with the highest severity. (4) Scoring was performed on every tooth. Results were categorized as either success (PAI 1 or 2) or failure (PAI 3 to 5). The success rate represented the percentage of cases with a successful outcome.

The CBCT‐PAI, proposed by Estrela et al. based on criteria established from measurements of periapical radiolucency interpreted on CBCT scans, was used for CBCT evaluation in this study (Estrela et al. 2008). The sizes of radiolucent images suggestive of periapical lesions were delimited and measured by using the working tools of Trophy DICOM software (version 6.4; Carestream Dental, Croissy‐Beaubourg, France) on CBCT scans in 3 dimensions: buccolingual, mesiodistal, and axial. The CBCT‐PAI was determined by the largest extension of the lesion. The worst PAI score was considered for multi‐rooted teeth. A 6‐point scoring system, including two additional variables (E for expansion of the cortical bone and D for destruction of the cortical bone), was used. The outcome of endodontic treatment was considered successful when the periapical area was scored as CBCT‐PAI ≤ 2. (Sălceanu et al. 2016, 2024) without any additional variables. Cases with a CBCT‐PAI score of 3, 4, or 5, or with evidence of cortical bone expansion (E) or destruction (D), were categorized as failures.

As this study was a retrospective, non‐interventional study, the radiographic image closest to the 1‐year follow‐up was selected, with the 1‐year point after the canal filling date serving as the reference. For long‐term outcome assessment, the most recent radiographic image available for follow‐up was evaluated. Three types of dental imaging were screened: full panoramic radiographs, periapical radiographs, and CBCT. Each type of image was assessed using the appropriate evaluation criteria: for periapical radiographs, the original PAI scoring system was applied; for CBCT, the CBCT‐PAI system was used; panoramic radiographs were evaluated for clear cases where the apical status could be determined, mainly to identify extreme cases (PAI 1, 4, or 5). When multiple images were available in close temporal proximity, the image with the highest resolution was selected. Success and failure were determined based on the respective criteria for each imaging modality.

### 2.6 Statistical Analysis

Data collected for AD patients and controls were entered into an Excel spreadsheet for comparison. Additionally, comparisons were performed between the AD subgroups: IBD, RA, and Ps. Statistical analysis of all data was performed using IBM SPSS Statistics for Windows, version 27.0 (IBM Corp., NY, USA). To assess the impact of the identified factors on the success rate, chi‐square tests using generalized estimation equations (GEE) were employed. Additionally, multivariable logistic regression analysis was performed to evaluate factors influencing the success rate of RCT in the study population. The significance level was established at 5% (α = 0.05). Odds ratios (OR) and 95% confidence intervals (CI) were obtained.

## 3 Results

### 3.1 Demographic Data of the Study

After application of a PSM system and exclusion criteria, the study included a total of 406 patients (203 in AD group and 203 in the control group) and 629 teeth (317 in AD group and 312 in the control group). Among the patients, 40 were female (9.85%) and 366 were male (90.15%). At the tooth level, 573 male teeth (91.09%) and 56 female teeth (8.91%) were involved. The mean age of the participants adjusting the number of teeth was 72.03 years, with a range of 69 to 76 years. The study group consisted of 317 teeth, while the control group comprised 312 teeth. Of the teeth, 147 (23.19%) were associated with IBD, 73 (11.51%) with RA, and 97 (15.3%) with Ps. Panoramic radiographs constituted the majority among the three radiographic modalities assessed. Preoperative periapical lesions were observed in 33% of the control group and 37% of the study group. The quality of root canal fillings was observed adequate in most cases.

### 3.2 Evaluation of 1‐Year Success Rate

Table 3 presents the 1‐year success rate of RCT in the study. RCT showed a 1‐year success rate of 73.72% for controls and 69.43% for the AD group. No significant difference was observed between the two groups. Within the AD patient group, the success rates were 64.63% for IBD, 75.34% for RA, and 73.20% for Ps. The success rate for the IBD group was the only one that demonstrated a statistically significant difference when compared with the control group (*p* = 0.025).

**TABLE 3:** 1‐Year success rate of root canal treatment in the study.

	Success, *N* (%)	Failure *N* (%)	Total	*p*
Control	230 (73.72)	82 (26.28)	312 (100)	
AD	221 (69.43)	96 (30.57)	317 (100)	0.268
IBD	95 (64.63)	52 (35.37)	147	0.025*
RA	55 (75.34)	18 (24.66)	73	0.49
Ps	71 (73.20)	26 (26.80)	97	0.50
Total	451 (71.57)	178 (28.43)		

Abbreviations: AD, autoimmune disease; IBD, inflammatory bowel disease; Ps, psoriasis; RA, rheumatoid arthritis.

* *p* < 0.05.

### 3.3 Evaluation of Long‐Term Success Rate

To analyse long‐term evaluation, the most recent dental clinic visit was recorded and evaluated. The observation period of each group was statistically calibrated to make a relevant comparison. Table 4 shows the long‐term success rate of RCT in the sample. The mean observation duration was approximately 50 months (1593.03 ± 1073.35 days), corresponding to nearly 4 years. The long‐term success rates of RCT for the control group and the autoimmune disease (AD) group were 66.03% and 61.51%, respectively (*p* = 0.207). Additionally, no significant difference was found among the three autoimmune disease subgroups (IBD = 59.18%; RA = 67.12%; Ps = 60.2%).

**TABLE 4:** Long‐term success rate of root canal treatment in the study.

	Success, *N* (%)	Failure, *N* (%)	Total	*p*
Control	206 (66.03)	106 (33.97)	312 (100)	
AD	195 (61.51)	122 (38.49)	317 (100)	0.207
IBD	87 (59.18)	60 (40.81)	147	0.125
RA	49 (67.12)	24 (32.88)	73	0.66
Ps	59 (60.82)	38 (39.18)	97	0.96
Total	401 (71.57)	228 (28.43)		

*Note:* Mean observation duration 1593.03 ± 1073.35 days.

Abbreviations: AD, autoimmune disease; IBD, inflammatory bowel disease; Ps, psoriasis; RA, rheumatoid arthritis.

### 3.4 Factors Affecting Success Rate

A multivariable logistic regression analysis was performed to identify the independent factors affecting the 1‐year success rate (Table 1, 5). The analysis revealed that arch and tooth type (OR, 1.12; 95% CI, 1.011–1.239; *p* = 0.029) and the presence of a preoperative periapical lesion (OR, 3.182; 95% CI, 2.228–4.544; *p* < 0.001) were significant predictors of treatment success. While the quality of root canal filling showed a tendency toward a positive association, it did not reach statistical significance (OR, 1.833; 95% CI, 0.954–3.521; *p* = 0.069). Notably, the presence of autoimmune disease was not identified as a significant independent factor influencing the success of RCT in this multivariable model (*p* > 0.05).

**TABLE 5:** Results of logistic regression analysis of factors affecting 1‐Year success rate.

		B	*p*	Exp (B)
Step 1	Age	0.005	0.706	1.005
	Sex	−0.156	0.618	0.810
	Arch type and tooth type	0.114	0.029*	1.121
	AD	−0.126	0.882	0.882
	Lesion	1.143	< 0.001*	3.137
	Quality of filling	0.585	0.080	1.796
	Constant	−1.945	0.044*	0.143
Step 8	Arch type and tooth type	0.113	0.029*	1.120
	Lesion	1.158	< 0.001*	3.182
	Quality of filling	0.606	0.069	1.833
	Constant	−1.797	< 0.001*	0.166

Abbreviation: AD, autoimmune disease.

* *p* < 0.05.

### 3.5 A Comparison of the Success Rates at 1 Year and Long‐Term

**FIGURE 2:**
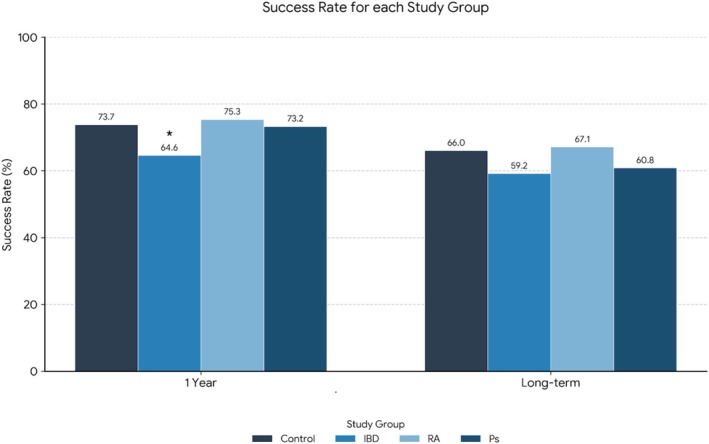
A comparison of the success rates at 1 year and long‐term. AD, autoimmune disease; IBD, inflammatory bowel disease, Ps, psoriasis; RA, rheumatoid arthritis; *p < 0.05.

Figure 2 shows success rate at 1 year and long‐term for control group and each autoimmune disease. Compared to the 1‐year results, a similar decreasing trend in long‐term outcomes was observed. The success rate in the control group declined from 73.72% at 1 year to 66.03% at long‐term follow‐up. In patients with IBD, the success rate showed a decline of approximately 5%, from 64.63% at 1 year to 59.18% over the long‐term follow‐up period. In RA patients, the success rate at 1 year was 75.34%, which declined by approximately 8% to 67.12% over the long‐term follow‐up period. Similarly, patients with Ps showed a decrease in success rate from 73.20% at 1 year to 60.82% in the long‐term follow‐up. However, there were no statistically significant differences observed between these values.

## 4 Discussion

The objective of this study was to explore whether autoimmune diseases affect the success of RCT. The null hypothesis tested—that there is no difference in RCT outcomes between control subjects and AD patients—was partially rejected. The result showed a positive relationship with the AP and IBD group (comparing the control) when measured at 1‐year post‐treatment. The other type of AD groups (RA, Ps) in this study showed no association between the prevalence of AP when evaluated at the 1‐year time point. In the long‐term outcome, there was no association found with any of the 3 diseases. However, logistic regression analysis indicated the presence of lesions, quality of root canal filling, and tooth location as significant factors, while any other autoimmune diseases were excluded.

Systemic health factors could potentially impact the success of RCT. In the present study, the success rate of autoimmune disease was slightly lower than the patient without autoimmune disease, although this difference was not significant. Several reports have explored the association between RCT outcomes and ADs (Barta 2020; Guerrero‐Girones et al. 2021). Given the involvement of similar immune cells, it is hypothesized that AP may be associated with AD. In the animal study, a decline in leukocyte levels was observed in immunosuppressed groups (Waterman Jr. et al. 1998). Given their weakened immunity, they are more prone to opportunistic infections, which could result in more severe consequences. Ng et al. (2008) also demonstrated that an impaired immune system had an impact on the healing of periapical tissues (Ng et al. 2008). Thus, pro‐inflammatory status and impaired immune response associated with systemic diseases can affect the reparative response of the dental pulp and periapical healing. Although there is no conclusive scientific evidence indicating that systemic diseases exert a significant influence on the success of RCT, the constrained study design necessitates cautious interpretation of even subtle trends, as these may reflect underlying biological effects.

One‐year outcomes of RCT show APs are more frequent in IBD patients. This finding generally is in accordance with previous reports addressing an association with IBD. Piras et al. reported that patients with IBD showed a higher prevalence of AP and increased PAI scores, particularly among female patients (Piras et al. 2017). Similarly, Poyato‐Borrego et al. observed that individuals with IBD were nearly six times more likely to develop AP than healthy controls (Poyato‐Borrego et al. 2021). In both studies AP was more frequent in endodontically treated teeth of patients with IBD compared to healthy individuals at the time of examination, irrespective of the duration since treatment. In addition, a case–control study indicated that patients with IBD underwent root canal fillings approximately four times more often than those without the disease (Poyato‐Borrego et al. 2020). These findings suggest that IBD could be a contributing risk factor for chronic inflammatory oral diseases such as periodontal disease and AP. In contrast, Segura‐Sampedro et al. found no notable difference in the occurrence of AP between individuals with IBD and control subjects (Segura‐Sampedro et al. 2022). Therefore, more research into the aetiology and risk factors of IBD is needed.

Both AP and AD are chronic inflammatory conditions that follow overlapping inflammatory mechanisms. During the development of AP, both the innate and adaptive immune systems contribute significantly, with particular emphasis on CD4+ T lymphocytes. Th1 cells produce interferon‐gamma (IFN‐γ) and tumour necrosis factor‐alpha (TNF‐α), which stimulate macrophage microbicidal functions and promote cell‐mediated immunity against intracellular pathogens. In contrast, Th2 cells release cytokines such as interleukin (IL)‐4, IL‐5, and IL‐13, which activate mast cells, eosinophils, and basophils, thereby regulating humoral immune responses (Wei et al. 2021). A sustained periapical immune response promotes osteoclast differentiation and inhibits osteogenesis, triggering and initiating periapical bone resorption. The imbalance between osteoclastogenesis and osteogenesis is the consequence of a combination of factors, with persistent bone destruction eventually evolving into chronic AP (Wen et al. 2024).

Ulcerative colitis is identified as a Th2 type immune disease, marked by increased expression of IL‐5, while Crohn's disease is considered a Th1 immune disease, characterized by elevated levels of IFN‐γ, IL‐12, and TNF‐α (Poyato‐Borrego et al. 2020). AP involves both types of immune responses. Initially, Th1 cells activate osteoclasts via the nuclear factor kappa B ligand (RANKL), leading to the characteristic destruction of periapical bone. To fully understand the impact of each disease and clarify the causal relationship, cross‐disciplinary collaboration between endodontists and immunologists is crucial.

The long‐term outcomes indicate that any of the diseases (IBD, RA, Ps) do not demonstrate statistical significance. The mean observation duration for long‐term outcomes was approximately 50 months. While IBD may have negatively affected the outcome at the 1‐year mark, its impact appears to diminish as time progresses. Generally, it takes at least 4 months for radiographic changes to become noticeable assuming a healthy adult with active bone metabolism. Considering the decreased rate of bone regeneration, this implies that it may take more than a year to confirm radiographic healing in IBD patients.

In all experimental groups included in the present study, the success rate of RCT was below 75%. According to a relevant systematic review on nonsurgical RCT (Ng et al. 2010), the estimated pooled proportion of teeth surviving 2 to 3 years after RCT, based on meta‐analyses, was 86.4% (95% CI: 74.7%–98.1%), indicating a higher tendency compared with the results of the present study. Several factors may account for the overall lower success rate observed in this study. First, 1 year after treatment is not a fully appropriate period to evaluate the success of RCT (Sabeti et al. 2024). Second, the use of CBCT in endodontics may contribute to a lower success rate when it comes to using conventional radiography. According to Estrela et al., AP was identified in nearly 40% of the cases by using periapical radiographs and in almost 61% of the cases by using CBCT scans (Estrela et al. 2008). As CBCT provides higher resolution, it is more accurate than periapical radiography for AP diagnosis. As the types of radiographic modalities used for assessment have evolved over time, this may have affected the results of the present study.

Also, a general trend of decreased success rates of RCT was observed in the long‐term outcomes compared to the 1‐year results. While the current findings did not reach statistical significance, the outcome may become significant with a larger sample size and an extended follow‐up period. Because the study population was confined to patients with the condition, the results may be influenced by as few as one or two cases.

The current study has some limitations. First, socioeconomic status and detailed pharmacological histories were not considered, which could act as potential confounders. In particular, the anti‐inflammatory nature of medications used to manage ADs such as corticosteroids, disease‐modifying antirheumatic drugs, and biological agents may significantly influence periapical healing. While some studies, such as Cotti et al. (Cotti et al. 2018), reported that IBD patients receiving anti‐TNF‐α therapy (e. g., adalimumab) exhibited faster periapical healing compared to healthy controls, our findings showed a different trend. This discrepancy might be attributed to the diversity in medication adherence, dosages, and the timing of therapy initiation among our study population. Due to the restricted access to integrated medical records from other departments in this retrospective design, we were unable to analyse the specific impact of medication types or dosages on RCT outcomes. Second, the inclusion of different radiographic modalities—periapical, panoramic, and CBCT imaging—presents a potential risk of diagnostic bias due to varying resolutions. However, this approach was adopted to ensure the representativeness of our 12‐year longitudinal cohort. In a long‐term retrospective study, excluding cases based solely on the imaging modality could result in significant selection bias, potentially excluding a large portion of the study population and compromising the statistical power. To mitigate the risk of inter‐modality discrepancies, we utilized these radiographs solely to determine a binary clinical outcome (Success vs. Failure) based on the clear presence or absence of radiolucency, rather than directly comparing raw scoring indices. Furthermore, all assessments were cross‐verified by two independent, calibrated examiners to ensure a conservative and reliable evaluation. Third, the patient population focused on specific gender and age groups. Due to the characteristics of the healthcare institution, the target population was inevitably biased, but they were mitigated as much as possible through PSM.

This study was designed and implemented with patients from a Veterans Health Service Medical Center (Seoul, South Korea). The investigation into the association between autoimmune diseases and endodontic treatment outcomes presents inherent challenges. Accurate analysis requires that patients receive both medical and dental care within the same medical institution. The acquisition of an adequate sample size is often limited by challenges related to patient follow‐up. As a central healthcare institution for national veterans, the hospital benefits from a highly loyal patient population with a strong tendency for regular visits. Furthermore, both medical and dental records are comprehensively documented and readily accessible, facilitating data integration and enabling the feasibility of this study. The distinct advantage of providing integrated medical and dental services within a single institution allowed for the collection of a sufficiently large sample size, representing a major strength of the present study.

## 5 Conclusion

Although initial bivariate analysis suggested a lower success rate in IBD patients, multivariable logistic regression revealed that the presence of autoimmune disease was not an independent predictor of treatment outcomes. Instead, the presence of preoperative periapical lesions and tooth type were identified as the primary significant determinants of success. Overall, these findings suggest that clinical factors play a more decisive role in determining success than the systemic autoimmune condition itself. Adhering to high‐quality endodontic protocols remains essential for optimizing outcomes in patients with autoimmune diseases.

## Author Contributions

Lee, Seoyeon: writing – original draft preparation, data curation, investigation, resources, visualization. Kim, Euiseong: writing – review and editing, conceptualization, supervision. Jung, Hoiin: writing – review and editing. Kim, Hyejin: methodology, resources, validation. Kim, Sunil: writing – review and editing, conceptualization, supervision, project administration, finding acquisition.

## Funding

This work was supported by the National Research Foundation of Korea (NRF) grant funded by the Korea government (MSIT) (No. RS‐2023‐00251473).

## Ethics Statement

This study was a non‐interventional clinical trial conducted under a standardized non‐experimental protocol. Accordingly, Institutional Review Board (IRB) approval was officially obtained (IRB file No. 2025–03‐001).

## Conflicts of Interest

The authors declare no conflicts of interest.

## Supporting information


**Data S1:** STROBE Statement—checklist of items that should be included in reports of observational studies.

## Data Availability

The data that support the findings of this study are available from the corresponding author upon reasonable request.
